# State T21, Restrictions on Flavored E-Cigarette Products, and Non-Medical Cannabis Sales Legalization in Relation to Young Adult Reports of Vape Shop Age Verification and Product Offerings: A Multilevel Analysis

**DOI:** 10.3390/ijerph192215079

**Published:** 2022-11-16

**Authors:** Zongshuan Duan, Yan Wang, Katelyn F. Romm, Lisa Henriksen, Nina C. Schleicher, Carla J. Berg

**Affiliations:** 1 Department of Prevention and Community Health, Milken Institute School of Public Health, George Washington University, Washington, DC 20052, USA; 2 TSET Health Promotion Research Center, Department of Pediatrics, College of Medicine, University of Oklahoma Health Sciences Center, Oklahoma City, OK 73104, USA; 3 Stanford Prevention Research Center, Department of Medicine, Stanford University School of Medicine, Palo Alto, CA 94304, USA; 4 George Washington Cancer Center, George Washington University, Washington, DC 20052, USA

**Keywords:** tobacco 21, flavor ban, non-medical cannabis, vape shop, age verification

## Abstract

Vape shop practices related to age verification and product offerings (e.g., other tobacco, cannabis), which may affect young-adult tobacco/substance use, are likely impacted by state-level policies (i.e., Tobacco 21 [T21], flavored e-cigarette restrictions, non-medical cannabis legalization). Using data from young adults (18–34 years) in 6 US states representing variability in whether/when they implemented the aforementioned policies, this study focused on past 6-month e-cigarette users who visited vape shops (Wave 1 [W1]: September–December 2018, *n* = 1127; W2: September–December 2019, *n* = 702; W3: September–December 2020, *n* = 549). Multilevel modeling examined T21 in relation to participants’ reports of age verification at last vape shop visit (among those < 27), and flavor restrictions and cannabis legalization in relation to noticing other tobacco or cannabis products at last visit. At W1–W3, 69.7%, 78.7%, and 75.8% of participants < 27 reported age verification, and participants increasingly noticed other tobacco (W2: 36.9%; W3: 48.6%) and cannabis products (W1: 25.8%; W2: 41.3%; W3: 58.3%). State T21 was unrelated to age verification (aOR = 1.19, 95%CI = 0.80–1.79); flavored e-cigarette restrictions correlated with noticing other tobacco products (aOR = 1.96, 95%CI = 1.10–3.51); flavored e-cigarette restrictions (aOR = 2.26, 95%CI = 1.57–3.24) and cannabis legalization (aOR = 2.84, 95%CI = 1.78–4.51) correlated with noticing cannabis products. Regulatory efforts must be informed by ongoing surveillance of such policies and their impact.

## 1. Introduction

The past decade marked substantial changes in the retail marketplace and regulatory environment for tobacco and cannabis products in the United States (US). The tobacco product landscape continues to diversify, featuring a variety of emerging or reemerging products [1], including e-cigarettes, smokeless tobacco (e.g., nicotine pouches) [2,3], and heated tobacco products (e.g., IQOS by Phillip Morris) [4,5], as well as co-use with cannabis products [6]. E-cigarettes are particularly prominent in the US market and are often distributed through tobacco specialty stores. Vape shops are tobacco specialty stores that exclusively sell e-cigarettes; they differ from smoke shops, which also sell other tobacco products [7]. Vape shops account for a meaningful proportion of retail e-cigarette sales (e.g., 19% in 2019) [8], and may serve as important sources of e-cigarette and tobacco-related information and influences on consumers’ perceptions [9,10]. In addition, tobacco specialty stores, including both vape shops and smoke shops, have become an increasingly prominent source for retail access and marketing of other tobacco products, as well as cannabis-derived products (i.e., cannabidiol [CBD], tetrahydrocannabinol [THC]) [11,12,13,14]. Given the potential impact of vape shops on consumers’ perceptions and use behaviors, the success of regulatory efforts depends on surveillance of vape shop practices, especially age verification and product offerings for other tobacco and cannabis products.

Tobacco 21 (T21) laws, which raise the minimum legal sales age from 18 to 21, have been shown to be effective in preventing tobacco initiation among youth and underaged adults [15,16,17,18,19,20]. As a result, the US government implemented the federal T21 law on 20 December 2019 [21], and prior to then, many US states and localities had implemented T21 laws. Although federal T21 declares no exemption of the minimum age requirement [21], there are gaps in enforcement protocols [22,23] and underestimates of noncompliance [24]. Particularly relevant to the current study, T21 implementation varies in relation to whether states previously had a T21 law in place [23,24] and whether state laws previously had required age verification for consumers under 27 years old (as the federal law requires) [25]. In addition, there is growing concern regarding enforcement and compliance for newer tobacco products, particularly e-cigarettes, particularly when they are sold online [26,27] or at specialty retailers like vape shops [27,28].

In January 2020, the US Food and Drug Administration (FDA) restricted sales of cartridge-based e-cigarettes that contained characterizing flavors other than menthol; notably, this ban did not extend to other types of e-cigarettes like mod-based systems or disposables, among other variations of e-cigarette devices [29]. Due to the critical exemptions of flavors and device types in the federal ban, states and many local jurisdictions have adopted more stringent restrictions on flavored e-cigarettes to close loopholes in the federal law [30]. As of 18 March 2022, 5 states and more than 300 localities enacted more comprehensive restrictions to reduce the availability of flavored e-cigarette products, and these numbers are likely to grow [31,32]. A study using market-level aggregated e-cigarette retail data from 2014 to 2020 showed that statewide restrictions on flavored e-cigarettes were associated with reductions in e-cigarette sales [33]. Perhaps related to increased restrictions on the e-cigarette market, evidence suggests that a significant proportion of vape shops have turned to selling other tobacco and cannabis products [11].

As tobacco regulation increases, an increasing number of states and districts have legalized sales of non-medical cannabis sales for adults age 21 or older [34,35]. Legalizing sales of non-medical cannabis increases its retail availability, which may influence consumers’ risk perceptions, including diminished harm perceptions towards cannabis use and increased perceived accessibility and availability of cannabis products [36,37]. In addition, such legalization may impact tobacco retail, as vape shop owners/managers may hold mixed attitudes towards the growing retail cannabis market [14], and an increasing proportion of vape shops offer or plan to offer cannabis products [11,12]. A recent study showed that approximately 80% of vape shop merchants sell CBD products, and the majority are interested in selling THC products [11].

Given the rapidly changing regulatory landscape, it is important to examine the potential impact of recent state regulatory policies (i.e., T21, restrictions on flavored e-cigarette products, legal sales of non-medical cannabis) on vape shop practices, which may affect consumer perceptions and use behaviors. This study analyzed data from a longitudinal online survey of US young adults from 6 metropolitan statistical areas (MSAs) conducted in 2018–2020. Drawing on the subset of participants who reported e-cigarette use and having visited vape shops, we examined: (1) change over time in customers’ reports of age verification and noticing tobacco/cannabis products; (2) the association of state T21 with age verification; and (3) the association of state flavored e-cigarette sales restrictions and legalization of non-medical cannabis sales on participants noticing other tobacco and cannabis products in vape shops.

## 2. Materials and Methods

### 2.1. Study Design and Participants

This study analyzed longitudinal data from a subset of young adults participating in the Vape shop Advertising, Place characteristics and Effects Surveillance (VAPES) study. This cohort study aims to examine vaping products’ retail environment and estimate its impacts on tobacco and substance use among US young adults from 6 MSAs, including Atlanta, Georgia; Boston, Massachusetts; Minneapolis, Minnesota; Oklahoma City, Oklahoma; San Diego, California; and Seattle, Washington. These MSAs were selected for their variations in state policies regarding tobacco and cannabis products (see Table 1 for an overview of state and federal policies in effect across waves) [38]. The study received ethical approval from the Emory University Institutional Review Board.

The detailed study design and data collection procedures are published elsewhere and briefly summarized here [39]. In fall (September–December) 2018, potential participants were recruited via social media (i.e., Facebook and Reddit), with enrollment criteria including: (1) living in one of the 6 MSAs; (2) 18–34 years old; and (3) English speaking. Purposive, quota-based sampling was used to ensure sufficient representation of e-cigarette and cigarette users (~1/3 each), roughly equal numbers of men and women, and 40% racial/ethnic minority [39].

Our sample was restricted to participants who reported any e-cigarette use in the past 6 months at each annual assessment, and endorsed visiting a vape shop (i.e., “In your [lifetime at Wave 1 [W1]; past year at W2 and W3], how many times have you visited a vape shop?”). This subsample included: at W1, *n* = 1127 (37.5% of 3006 total W1 participants); at W2, *n* = 702 (29.6% of 2375 W2 participants); and at W3, *n* = 549 (22.2% of 2476 W3 participants). Therefore, current analyses were restricted to 1339 participants who reported past 6-month e-cigarette use and also reported ≥1 vape shop visits for at least one annual assessment.

### 2.2. Measures

#### 2.2.1. Independent Variables

*State policies:* For analyses examining age verification as an outcome, the primary predictor was the *existence of a state-level T21 law* across waves (see Table 1). At W1, California was the only state with T21 (effective 6/9/2016); Massachusetts adopted by state T21 by W2 (effective 31 December 2018); state T21 was adopted by W3 in Washington (effective 1 January 2020), Minnesota (effective 1 August 2020), and Oklahoma (effective 19 May 2020) [40]. Georgia did not adopt a state T21 law by fall 2020. In 2018–2020, few local jurisdictions in this study had implemented T21 laws, including Boston (2016) and Minneapolis (2018) [40]. Between W2 and W3, the federal T21 law went into effect [21].

For analyses examining the outcomes related to noticing other tobacco or cannabis products, the primary predictors were the existence of *state-level restrictions on flavored e-cigarettes* and *legal non-medical cannabis sales*. In November 2019, Massachusetts became the first state to restrict the sale of flavored tobacco products (effective 27 November 2019 for e-cigarettes and 1 June 2020 for other tobacco products) [41]. In response to e-cigarette, or vaping, product use associated lung injury (EVALI), Washington temporarily restricted flavored e-cigarette sales in fall 2019 (effective October 2019–February 2020) [41]. Note that, in 2018–2020, few local jurisdictions in the study areas had flavor restrictions, with only a few in California, Massachusetts, and Minnesota (but none in Georgia, Oklahoma, or Washington) [41].

Regarding state legalization of non-medical cannabis sales, these were legalized before W1 in Washington (effective 8 July 2014) and California (effective 1 January 2018); Massachusetts legalized non-medical cannabis sales between W1 and W2 (effective 20 November 2018) [42].

#### 2.2.2. Outcomes

*Age verification*: At W1–W3, participants were asked, “The last time you visited a vape shop, did you get asked for identification to verify your age upon: (1) entering a vape shop; and (2) making a purchase?” These questions were asked because some states (e.g., Washington) define vape shops as adult-only establishments; thus, any purchase made at a vape shop would require identification checks prior to entry (and therefore purchase). Participants who reported “yes” to either question were categorized as experiencing age verification upon entering or purchasing. Although these questions were asked among all participants, analyses regarding age verification only included participants under the age of 27 to align with FDA regulations regarding required age verification: W1, *n* = 837; W2, *n* = 459; and W3, *n* = 326 [21]. To further characterize vape shop settings, we also assessed whether participants noticed age requirements signage.

*Noticing other tobacco and cannabis products:* At W2 and W3, participants were asked, “The last time you visited a vape shop, did you notice: (1) new tobacco products that heat but do not burn tobacco, like Eclipse or IQOS; and (2) other tobacco products, such as cigarettes, cigars, or hookahs?” Participants who reported “yes” to either question were coded as noticing other tobacco products.

Similarly, at W1–W3, participants were asked, “The last time you visited a vape shop, did you get told or notice: (1) e-cigarettes were available for use to vaporize marijuana (CBD and/or THC); (2) the vape shop had e-liquids that contain marijuana (CBD and/or THC); and (3) the vape shop had other products (for example, edibles) that contain marijuana (CBD and/or THC)?” Those who reported “yes” to any question were coded as noticing cannabis products.

#### 2.2.3. Descriptive Variables and Covariates

*Other vape shop characteristics:* At W1–W3, participants asked, “The last time you visited a vape shop, did you: get offered free samples; get offered price promotions; get told that e-cigarettes help smokers quit smoking; get told that e-cigarettes are safer than regular cigarettes; get told that e-cigarettes have no health risks; and notice health warning labels on products and ads”.

*Covariates:* Sociodemographic factors (from W1) included: age, sex (male/female), sexual orientation (heterosexual/sexual minority), race (White/Black/Asian/other), and ethnicity (Hispanic/non-Hispanic).

### 2.3. Data Analysis

Using Stata 15.1 (StataCorp, College Station, TX, USA), univariate analyses were conducted to describe outcomes, exposures, and covariates. Multilevel logistic regression models compared the differences in outcomes of 2 consecutive waves and estimated trends in percentages over the 3 waves, using wave as a continuous variable. These multilevel models were fitted using a random intercept at participant level to account for repeated measures within individuals and a random intercept at the MSA level to account for clustering of individuals within MSAs.

Additionally, multilevel models examined: (1) state T21 status in relation to participants’ reports of age verification at last vape shop visit (among those < 27); (2) flavor restrictions in relation to noticing (a) other tobacco and (b) cannabis products; and (3) cannabis legalization in relation to noticing (a) other tobacco and (b) cannabis products, adjusting for sociodemographics (i.e., age, sex, sexual orientation, race, ethnicity). Each state policy was coded as a time-variant predictor for each time point. Given that Washington had an emergency policy to temporarily restrict flavored e-cigarette sales in fall 2019 (effective October 2019–February 2020), we constructed 2 multilevel logistic regression models to estimate the association between outcomes and state restrictions on flavored e-cigarettes with and without Washington’s temporary ban at W2 for sensitivity analysis. Missing values were handled using maximum likelihood methods, assuming missingness was at random [43]. All statistical tests were two-sided with the significance level set to α = 0.05.

## 3. Results

### 3.1. Participant Characteristics

As shown in Table 2, the overall W1 sample included 1339 participants (1127 at W1, 702 at W2, and 549 at W3). At W1, the sample was 24.08 years old on average, 74.3% under age 27, 51.9% female, 36.1% sexual minority, 75.9% White, and 14.1% Hispanic.

### 3.2. State T21 in Relation to Age Verification

Participants living in states with T21 represented 14.9%, 33.5%, and 84.7% of the sample at W1, W2, and W3, respectively (Table 2). As indicated in Table 3, among those under 27, the percent confirming age verification upon entering or purchasing was 69.7% at W1, 78.7% at W2, and 75.8% at W3 (*p* = 0.053 for trend; see also Supplemental Figure S1a).

In regression models controlling for age, sex, sexual orientation, and race/ethnicity (Table 4), state T21 was not significantly associated with age verification upon entering or purchasing at the last visit (aOR = 1.19, 95%CI = 0.80–1.79). No covariates were correlated.

### 3.3. State Flavored E-Cigarette Restrictions and Legal Cannabis in Relation to Noticing Other Tobacco and Cannabis Products

At W1–W3, the proportions of the sample representing states with e-cigarette flavor restrictions were: 0%, 16.8% [categorizing Washington as having restrictions], and 33.5%, respectively; and the proportions representing states with non-medical cannabis sales were: 36.0%, 50.4%, and 50.7% (Table 2). The percent who noticed other tobacco products in vape shops (only assessed at W2 and W3) increased from W2 (36.9%) to W3 (48.6%, *p* < 0.001; Table 3), including heated tobacco products (3.8% at W2 and 11.7% at W3, *p* = 0.001) and other tobacco products (35.8% at W2, 43.7% at W3, *p* < 0.001; see also Supplemental Figure S1b). There was a significant increase in noticing cannabis-derived products at last vape shop visit over the study period (25.8% at W1, 41.3% at W2, 58.3% at W3; *p* < 0.001 for trend), including e-cigarettes for vaping CBD or THC (18.6% at W1, 28.9% at W2, 40.1% at W3; *p* < 0.001), e-liquids (13.0% at W1, 17.1% at W2, 40.8% at W3; *p* < 0.001), and other products (13.2% at W1, 25.4% at W2, 33.2% at W3; *p* < 0.001; Supplemental Figure S1c).

In regression models (Table 4), state restrictions on flavored e-cigarettes were associated with significantly higher odds of noticing tobacco products (aOR = 1.96, 95%CI = 1.10–3.51) and noticing any cannabis products (aOR = 2.26, 95%CI = 1.57–3.24) in the model including Washington as a state with e-cigarette restrictions at W2; the model excluding Washington as having restrictions indicated similar but stronger associations. State legalization of non-medical cannabis sales was associated with higher odds of noticing any cannabis product (aOR = 2.84, 95%CI = 1.78–4.51), but not with noticing other tobacco products (aOR = 0.63, 95%CI = 0.29–1.37). In both models, older age was associated with lower odds of noticing other tobacco products, and being older and female was associated with higher odds of noticing any cannabis product.

### 3.4. Other Vape Shop Experiences

As noted in Table 3 and Supplemental Figure S1d, there were downward trends in exposure to promotional tactics (i.e., offered free samples, price promotions), relatively stable trends for exposure to health claims (i.e., being informed that e-cigarettes help smokers quit smoking, e-cigarettes are safer than regular cigarettes, e-cigarettes have no health risks), and an upward trend in noticing health warning information (i.e., health warning labels on products and ads).

## 4. Discussion

This study provides unique data regarding multi-year trends in vape shop practices, as experienced by a multi-state cohort of e-cigarette users. Trend analyses indicated statistically significant increases in the proportion of young adult e-cigarette users under age 27 who reported being asked for age verification during their last vape shop visit from 2018 to 2019, consistent with a study of state T21 laws using national surveillance data [44]. However, whether this trend is practically significant is questionable, and particularly concerning is the limited change from 2019 to 2020 after the federal T21 went into effect. This may be in part due to public health systems responsible for tobacco policy compliance being under stress during the COVID-19 pandemic. E-cigarette users in this study also indicated increasingly noticing other tobacco and cannabis products in vape shops, which aligns with findings from previous retail marketing surveillance [11,12]. Additionally, current findings suggest downward trends in exposure to promotional tactics and health claims, and upward trends in health warning information [11,12,13,14]. These findings are critical to inform future surveillance of the vape shop retail environment, and have important implications for federal, state, and local authorities to improve compliance of relevant policies.

Multilevel modeling also examined the associations between relevant state-level policies (e.g., T21, e-cigarette flavor restrictions, non-medical cannabis sales) and participant experiences of vape shop practices. Notably, findings from these analyses indicated that state T21 was not significantly associated with age verification upon entering vape shops or purchasing e-cigarettes at vape shops among participants under age 27, suggesting potential variations in the implementation and enforcement of T21 laws across states [23,24]. This may also be partially explained by the fact that state T21 laws varied in terms of guidance on the minimum age for verification [45,46]. For instance, this age ranges from 21 to 30 years old across states, and multiple states (e.g., California, Georgia, Oklahoma, Washington) do not require age verification for purchasers who appear older than 21 [25]. This finding suggests that variations in regulations and noncompliance may undermine the potential impact of increasing the minimum legal sales age [15,16,17].

Findings also indicated specificity in terms of the effects of state restrictions on flavored products and cannabis retail legalization. More specifically, living in a state with e-cigarette flavor restrictions was associated with noticing other tobacco and cannabis products available in vape shops, while living in a state with legalized non-medical cannabis retail was associated with increases in noticing cannabis products offered in vape shops. Given that 2 of the 6 states that legalized non-medical cannabis sales also had the most stringent state or local restrictions on flavored e-cigarettes, it is not surprising that restrictions on flavored e-cigarette products were also associated with increased availability of cannabis products in vape shops. Given the anticipated increases in flavored e-cigarette restrictions [29,47], vape shops may increase the availability of other types of tobacco products [11,12,13,14] and sell cannabis products where allowed [11].

Additionally, results showed that older age was associated with noticing cannabis-derived products at the last vape shop visit, but inversely associated with noticing other tobacco products. Older young adults may be more aware of cannabis products and have more conditions to use these products [48], while younger people may be more likely to be attracted to other tobacco products (e.g., heated tobacco products) typically featuring innovative designs [4,49,50,51,52]. In addition, female e-cigarette users were more likely to notice cannabis-derived products relative to their male counterparts, a finding that requires further investigation.

Current findings have implications for regulatory efforts and future research. First, findings that state T21 laws were not associated with age verification practices and the minimal changes in age verification rates during the implementation of the federal T21 indicate the need for surveillance to examine enforcement protocols and their effects. Second, results indicated that increased restrictions of e-cigarette products may prompt vape shops to expand their product offerings to include other tobacco and/or cannabis, which could result in unintended consequences (e.g., undermine efforts by consumers to quit combustible tobacco use), given the role of vape shops’ in shaping consumers’ perceptions [9,10]. In addition, the effectiveness of restrictions on flavored products may be further compromised by retailers’ noncompliance [11]. Future research, particularly examining the effects of different types of restrictions on flavored tobacco products (e.g., partial ban, comprehensive ban, different flavors or device types) at state and local levels, is needed to provide a more complete picture of how retailers and consumers may react to different restrictions.

Study limitations include the use of self-report survey measures, which are subject to recall bias and social desirability bias [53]. Relatedly, the survey did not explicitly define vape shops, so participants may have differentially interpreted the types of retailers that were being assessed (e.g., vape shops that sell e-cigarettes but not other tobacco vs. smoke shops that sell both). Additionally, survey assessments did not ask participants to specify which other tobacco products or cannabis-derived products they noticed at vape shops. There was also inconsistency in the measures used to assess age verification (i.e., past 6-month e-cigarette users who reported lifetime vape shop visit at W1 vs. past-year vape shop visit at W2 and W3). In addition, this study focused on T21, restrictions on flavored e-cigarettes, and non-medical cannabis sales, but did not control for exposure to local policies or include questions regarding the implications of other existing laws (e.g., the Prevent All Cigarette Trafficking Act [54]) or proposed regulation (e.g., increasing taxes for tobacco and cannabis products). Finally, findings from this non-probability sample of US young adults in 6 MSAs may not be generalizable to these MSAs or other regions in the US, and attrition across waves may undermine internal validity. Nonetheless, current findings provide insights that can be further investigated by future studies focusing on vape retail settings with a probability sample.

## 5. Conclusions

Current findings highlight potential complexities in the impacts of state regulations (i.e., T21, restrictions on sales of flavored e-cigarette products, and legalizing non-medical cannabis sales) on vape shop practices, including age verification and tobacco and cannabis product offerings as reported by e-cigarette users. Continued surveillance of vape shop practices is warranted, particularly given the rapidly changing tobacco landscape and regulatory environment.

## Figures and Tables

**Table 1:** Timeline of state and federal e-cigarette related policies (i.e., T21, restrictions on flavored e-cigarettes, and legalized non-medical cannabis sales) in effect in fall 2018 (Wave 1), fall 2019 (Wave 2), and fall 2020 (Wave 3).

	Wave 1	Wave 2	Wave 3
Survey Dates	September–December 2018	September–December 2019	September–December 2020
**State T21**			
California	Yes; Effective 9 June 2016	Yes	Yes
Massachusetts	No *	Yes; Effective 31 December 2018	Yes
Washington	No	No	Yes; Effective 1 January 2020
Minnesota	No	No	Yes; Effective 1 August 2020
Oklahoma	No	No	Yes; Effective 19 May 2020
Georgia	No	No	No
**State flavored e-cigarette restrictions**			
California	No	No	No
Massachusetts	No	No	Yes; Effective 27 November 2019
Washington	No	Yes; ^ Effective October 2019–February 2020	No
Minnesota	No	No	No
Oklahoma	No	No	No
Georgia	No	No	No
**State legalized non-medical cannabis sales**			
California	Yes; Effective 1 January 2018	Yes	Yes
Massachusetts	No	Yes; Effective 20 November 2018	Yes
Washington	Yes; Effective 8 July 2014	Yes	Yes
Minnesota	No	No	No
Oklahoma	No	No	No
Georgia	No	No	No
**Federal Policies**			
T21	No	No	Yes; Effective 20 December 2019
Flavored cartridge e-cigarette ban	No	No	Yes; Effective 1 February 2020
Non-medical cannabis	No	No	No

Notes: * Local T21 covered 66.7% state population in 2018. ^ Washington had an emergency rule to temporarily restrict flavored e-cigarette sales October 2019–February 2020.

**Table 2:** Characteristics of past 6-month e-cigarette users who visited vape shops *.

	Past 6-Month E-Cigarette Users		
	**Wave 1**	**Wave 2**	**Wave 3**
	***n* = 1127**	***n* = 702**	***n* = 549**
**Variables**	***n* (%) ^**	***n* (%) ^**	***n* (%) ^**
***State policy factors***			
T21	168 (14.9)	235 (33.5)	461 (84.7)
E-cigarette flavor restrictions (including Washington)	0 (0)	118 (16.8)	182 (33.5)
E-cigarette flavor restrictions (excluding Washington)	0 (0)	0 (0)	86 (15.8)
Non-medical cannabis	406 (36.0)	353 (50.4)	276 (50.7)
***Metropolitan statistical area***			
Atlanta	179 (15.9)	126 (18.0)	78 (14.3)
Boston	177 (15.7)	107 (15.2)	83 (15.2)
Minneapolis	231 (20.5)	145 (20.7)	113 (20.7)
Oklahoma City	134 (11.9)	70 (10.0)	61 (11.2)
San Diego	168 (14.9)	117 (16.7)	76 (13.9)
Seattle	238 (21.1)	118 (16.8)	95 (17.4)
Other	0 (0)	19 (2.7)	40 (7.3)
***Sociodemographics***			
Age (M/SD)	24.08 (4.72)	25.45 (4.81)	26.47 (4.90)
Age < 27 years old	837 (74.3)	459 (65.4)	326 (59.4)
Female ^§^	565 (51.9)	371 (54.0)	274 (51.4)
Sexual minority status	403 (36.1)	227 (32.8)	188 (34.6)
Race			
White	842 (75.9)	511 (74.2)	389 (71.8)
Black	42 (3.8)	29 (4.2)	27 (5.0)
Asian	105 (9.5)	69 (10.0)	66 (12.2)
Other	120 (10.8)	80 (11.6)	60 (11.1)
Hispanic	158 (14.1)	100 (14.3)	66 (12.0)

Notes: ^ All % are column %. * Reported visiting a vape shop in their lifetime at W1 or in the past year at W2 or W3. ^§^ Other: W1 *n* = 38, W2 *n* = 15, W3 *n* = 16.

**Table 3:** Vape shop practices over time reported by past 6-month e-cigarette users who visited vape shops *.

Experiences at Vape Shop at Most Recent Visit	Wave 1	Wave 2	Wave 3	*p*, W1–W2 Change	*p*, W2–W3 Change	*p*, W1–W3 Trend
	*n* (%)	*n* (%)	*n* (%)			
***Age verification—All participants***	***n* = 1127**	***n* = 702**	***n* = 549**			
*Asked for age verification upon entering or purchasing*	755 (67.0)	531 (75.6)	403 (73.4)	^§^	^§^	**<0.001**
Asked for age verification upon entering	417 (37.0)	276 (39.3)	199 (36.2)	0.272	0.116	0.752
Asked for age verification upon purchasing	621 (55.1)	446 (63.5)	341 (62.1)	**<0.001**	0.546	**<0.001**
*Noticed age requirement signs*	824 (73.1)	557 (79.3)	433 (78.9)	**<0.001**	0.79	**<0.001**
***Age verification—Young adults < 27 years old***	***n* = 837**	***n* = 459**	***n* = 326**			
*Asked for age verification upon entering or purchasing*	583 (69.7)	361 (78.7)	247 (75.8)	0.088	^§^	0.053
Asked for age verification upon entering	316 (37.8)	177 (38.6)	109 (33.4)	0.973	0.092	0.16
Asked for age verification upon purchasing	488 (58.3)	308 (67.1)	218 (66.9)	**0.001**	0.79	**0.001**
*Noticed age requirement signs*	642 (76.7)	370 (80.6)	259 (79.5)	0.072	0.621	0.072
***Noticed other tobacco and cannabis products***	***n* = 1127**	***n* = 702**	***n* = 549**			
*Noticed any other tobacco products*	--	259 (36.9)	267 (48.6)	**--**	**<0.001**	**--**
Noticed heated tobacco products	--	27 (3.8)	64 (11.7)	**--**	**0.001**	**--**
Noticed other tobacco products	--	251 (35.8)	240 (43.7)	**--**	**<0.001**	**--**
*Noticed any cannabis-derived products*	291 (25.8)	290 (41.3)	320 (58.3)	**<0.001**	**<0.001**	**<0.001**
Noticed e-cigarettes for vaping CBD and/or THC	210 (18.6)	203 (28.9)	220 (40.1)	**<0.001**	**<0.001**	**<0.001**
Noticed e-liquids containing CBD and/or THC	146 (13.0)	120 (17.1)	224 (40.8)	**0.01**	**<0.001**	**<0.001**
Noticed other products containing CBD and/or THC	149 (13.2)	178 (25.4)	182 (33.2)	**<0.001**	**0.001**	**<0.001**
***Promotions and warnings***	***n* = 1127**	***n* = 702**	***n* = 549**			
Offered free samples	180 (16.0)	84 (12.0)	64 (11.7)	**0.007**	0.889	**0.003**
Offered price promotions	379 (33.6)	193 (27.5)	171 (31.1)	**0.004**	0.179	0.073
Told e-cigarettes help smokers quit smoking	186 (16.5)	130 (18.5)	96 (17.5)	0.165	0.391	0.533
Told e-cigarettes are safer than regular cigarettes	152 (13.5)	102 (14.5)	78 (14.2)	0.626	0.603	0.865
Told e-cigarettes have no health risks	50 (4.4)	40 (5.7)	11 (2.0)	0.18	**0.002**	0.073
Noticed health warning labels on products and ads	513 (45.5)	427 (60.8)	435 (79.2)	**<0.001**	**<0.001**	**<0.001**

Notes: Bold indicates *p* < 0.05. * Reported visiting a vape shop in their lifetime at W1 or in the past year at W2 or W3. ^§^ Did not concave.

**Table 4:** Multilevel logistic regression results examining relevant state policies in relation to key vape shop related outcomes among past 6-month e-cigarette users who visited vape shops *.

	State T21	State Flavor Restrictions				State Non-Medical Cannabis	
	Age Verified to Enter or Buy ^#^	*Including Washington at W2*		*Excluding Washington at W2*		Noticed Other Tobacco Products ^	Noticed Any Cannabis Products ^§^
		Noticed Other Tobacco Products ^	Noticed Any Cannabis Products ^§^	Noticed Other Tobacco Products ^	Noticed any Cannabis Products ^§^		
Variable	aOR (CI)	aOR (CI)	aOR (CI)	aOR (CI)	aOR (CI)	aOR (CI)	aOR (CI)
***State policy factors***							
T21	1.19 (0.80–1.79)						
E-cigarette flavor ban		**1.96 (1.10–3.51)**	**2.26 (1.57–3.24)**	**2.70 (1.37–5.29)**	**5.76 (3.18–10.45)**		
Non-medical cannabis						0.63 (0.29–1.37)	**2.84 (1.78–4.51)**
***Sociodemographics***							
Age	1.03 (0.96–1.10)	**0.96 (0.93–0.99)**	**1.04 (1.02–1.06)**	**0.96 (0.93–0.99)**	**1.04 (1.02–1.06)**	**0.96 (0.93–0.99)**	**1.04 (1.02–1.06)**
Female (ref = male)	0.94 (0.65–1.35)	1.01 (0.74–1.38)	**1.45 (1.16–1.82)**	1.02 (0.74–1.39)	**1.46 (1.17–1.83)**	1.00 (0.74–1.36)	**1.46 (1.17–1.83)**
Sexual minority (ref = no)	0.85 (0.58–1.25)	1.06 (0.76–1.48)	1.08 (0.85–1.37)	1.06 (0.76–1.49)	1.07 (0.85–1.36)	1.08 (0.78–1.49)	1.09 (0.86–1.38)
Race (ref = White)							
Black	1.18 (0.36–3.89)	1.02 (0.49–2.14)	0.81 (0.47–1.41)	1.01 (0.48–2.12)	0.81 (0.46–1.40)	1.03 (0.50–2.12)	0.78 (0.45–1.35)
Asian	1.37 (0.77–2.41)	0.82 (0.50–1.33)	1.19 (0.83–1.70)	0.84 (0.51–1.37)	1.21 (0.85–1.73)	0.88 (0.55–1.41)	1.19 (0.83–1.70)
Other	1.01 (0.56–1.80)	0.97 (0.58–1.62)	1.25 (0.87–1.78)	0.96 (0.58–1.60)	1.24 (0.87–1.77)	0.98 (0.59–1.60)	1.23 (0.86–0.75)
Hispanic (ref = no)	0.86 (0.50–1.47)	1.05 (0.65–1.68)	1.22 (0.87–1.70)	1.05 (0.66–1.70)	1.22 (0.88–1.69)	1.04 (0.65–1.65)	1.20 (0.86–1.67)
ICC							
MSA (*n* = 6)	0.012	0.078	0.007	0.083	0.024	0.094	0.086
Person	0.435	0.3	0.195	0.301	0.211	0.286	0.252

Notes: Bold indicates *p* < 0.05. CI: 95% Confidence Interval. ICC: Intraclass correlation. MSA: Metropolitan statistical area. * Reported visiting a vape shop in their lifetime at W1 or in the past year at W2 or W3. **^#^** Analyses restricted to those < 27 years old, *n* = 1528. ^ Assessed only at W2 and W3, *n* = 1.179. ^§^ *n* = 2240.

## Data Availability

Data not publicly available (available upon request).

## Supplementary Materials

The following supporting information can be downloaded at: https://www.mdpi.com/article/10.3390/ijerph192215079/s1, Figure S1: Vape shop practices over time among participants reporting past 6-month e-cigarette use and lifetime/past-year vape shop visits.
