# Supplementary material for: State T21, Restrictions on Flavored E-Cigarette Products, and Non-Medical Cannabis Sales Legalization in Relation to Young Adult Reports of Vape Shop Age Verification and Product Offerings: A Multilevel Analysis
Source: Int J Environ Res Public Health. 2022 Nov 16;19(22):15079. doi: 10.3390/ijerph192215079 (PMC9690108; doi:10.3390/ijerph192215079)
Supplement: Supplementary file 1 [file ijerph-19-15079-s001.zip › ijerph-1982682-supplementary.pdf]

Supplemental Figure S1. Vape shop practices over time among participants reporting past 6-month e-cigarette use and lifetime/past-year vape shop visits.

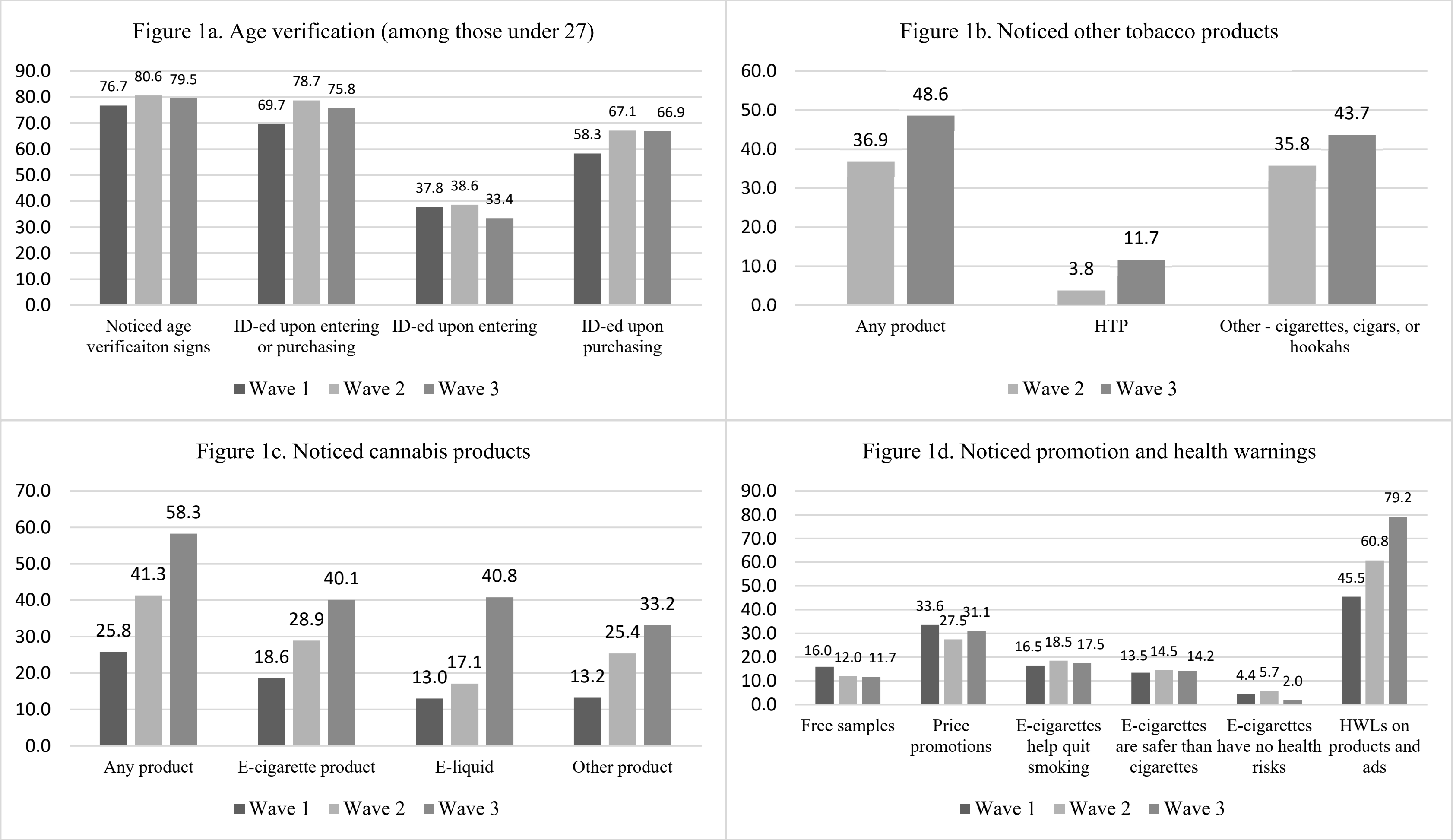

Notes: Figure 1a, significant differences between W1 and W2 for noticing age verification signs and being asked for age verification upon entering/purchasing and for purchasing specifically. Figure 1b, significant differences between W2 and W3 for noticing any other tobacco products (including HTPs and others, respectively). Figure 1c, significant differences between W1 and W2 and between W2 and W3 for noticing any product related to CBD or THC (and for each specific item, respectively). Figure 1d, significant differences between W1 and W2 offered free samples, offered price promotions, noticed health warning labels on products and ads, and between W2 and W3 told e-cigarettes have no health risks.
